# The Emerging Role of Gut Microbiota in Myalgic Encephalomyelitis/Chronic Fatigue Syndrome (ME/CFS): Current Evidence and Potential Therapeutic Applications

**DOI:** 10.3390/jcm10215077

**Published:** 2021-10-29

**Authors:** Angelica Varesi, Undine-Sophie Deumer, Sanjana Ananth, Giovanni Ricevuti

**Affiliations:** 1 Department of Biology and Biotechnology, University of Pavia, 27100 Pavia, Italy; 2 Almo Collegio Borromeo, 27100 Pavia, Italy; 3 Department of Biological Sciences, Faculty of Natural Sciences and Mathematics, University of Cologne, 50674 Cologne, Germany; udeumer@small.Uni-Koeln.de; 4 Department of Metabolism, Digestion and Reproduction, Faculty of Medicine, Imperial College London, London SW7 2AZ, UK; s.ananth@imperial.ac.uk; 5 Department of Drug Sciences, School of Pharmacy, University of Pavia, 27100 Pavia, Italy

**Keywords:** ME/CFS, dysbiosis, therapy, diagnosis, intestinal permeability, metabolic endotoxemia, LPS

## Abstract

The well-known symptoms of Myalgic Encephalomyelitis/Chronic Fatigue Syndrome (ME/CFS) are chronic pain, cognitive dysfunction, post-exertional malaise and severe fatigue. Another class of symptoms commonly reported in the context of ME/CFS are gastrointestinal (GI) problems. These may occur due to comorbidities such as Crohn’s disease or irritable bowel syndrome (IBS), or as a symptom of ME/CFS itself due to an interruption of the complex interplay between the gut microbiota (GM) and the host GI tract. An altered composition and overall decrease in diversity of GM has been observed in ME/CFS cases compared to controls. In this review, we reflect on genetics, infections, and other influences that may factor into the alterations seen in the GM of ME/CFS individuals, we discuss consequences arising from these changes, and we contemplate the therapeutic potential of treating the gut to alleviate ME/CFS symptoms holistically.

## 1. Introduction

Since the late 19th century, reasonably reliable medical records have been available which describe a multisystemic and debilitating disease of unknown origin causing chronic and severe fatigue which prevents individuals from carrying out normal levels of day-to-day activities [1]. Today, this disease is known under the terms myalgic encephalomyelitis and chronic fatigue syndrome (ME/CFS) and is diagnosed based on symptoms using established consensus criteria (i.e., Fukuda, Canadian Consensus Criteria, Oxford, International Consensus Criteria, etc.) [2,3,4,5]. Besides disabling fatigue, cognitive dysfunction, sleep problems, autonomic dysfunction, and post-exertional malaise are often reported in individuals with ME/CFS [6]. While ME/CFS is clearly accompanied by immunological alterations and inflammatory dysfunctions [7,8,9,10,11,12], recent findings suggests that a link between microbial dysbiosis and disease pathogenesis is also possible [13,14,15]. Although the precise etiology of ME/CFS is poorly understood, genetic predisposition, viral infection, and stress have been considered to be linked with disease origin and chronicity [6,16,17,18]. For example, the finding that relatives of ME/CFS cases report significantly higher rates of ME/CFS or similar fatigue-like symptoms compared to random controls may indicate a genetic contribution to disease onset [19,20,21]. However, independent studies on different cohorts often lack reproducibility, thus evidencing the need for new larger investigations [22]. Similarly, pathogens such as Epstein-Barr Virus (EBV), Human Herpesvirus (HHV)-6, and Human Parvovirus B19 are suspected of contributing to the development of the disease via antiviral immune activation and systemic inflammation [23,24,25,26,27,28,29], but their necessity for ME/CFS development remains debated [30]. Indeed, several studies comparing ME/CFS cases with controls failed to support the hypothesis of involvement of a viral infection in disease pathogenesis [29,31,32,33,34,35]. Moreover, it should be noted that the vast majority of people recover from infections without consequences, therefore making it difficult to establish a clear correlation between infection and ME/CFS. Other infectious diseases such as Lyme disease or COVID-19 have also been suggested to increase the risk of developing ME/CFS [36,37]; yet the mechanism behind this is largely unknown. One hypothesis is that the infection causes inflammation in the body, which dysregulates the immune response and inflammatory cascades in the long term [10,11,18,38]; but how this impacts the onset of ME/CFS has yet to be defined. 

The term “gut microbiota” (GM) describes the microbial community in the gastrointestinal (GI) tract, which consists of a plethora of bacteria, archaea, phages, yeasts, protozoa, and fungal species that exist in a symbiotic relationship with the human gut. Owing to advancements in genomic studies and metagenomic analysis, GM composition has been studied regarding development of certain diseases such as neuro-psychological disorders, cancer, cardio-metabolic disorders, and inflammatory bowel disease (IBD) [39,40]. *Firmicutes*, *Bacteroides*, *Proteobacteria*, *Fusobacteria*, *Verrucomicrobia*, *Cyanobacteria*, and *Actinobacteria* are the major taxonomic groups typically found in the gut [41,42]. As the GM and their habitat are involved in a complex interplay, host environmental factors such as pH, transit time, bile acids, digestive enzymes, and mucus play an important role in GM composition [42,43,44]. Non-host factors involved can be nutrients and medications, as well as bacterial properties such as adhesion, metabolic capacity, and enzymes [44,45]. The microbiota produces many chemical mediators that can travel to distant regions, such as the brain, and affect the host′s health positively or negatively [46,47]. Indeed, by synthesizing nutrients and vitamins, producing beneficial or toxic metabolites, inhibiting microbial and viral pathogens, detoxifying food, and contributing to the development of a healthy immune system, GM are essential for the host [42,43,44]. Depending on the GM composition, effects on the immune system can differ. Immune cell priming partly takes place in the gut and signals for the development of T regulatory, T helper (Th-1 and Th-2), and Th-17 cells are generated, which are involved in immune system regulation and cytokine secretion as a defense against foreign antigens [48,49,50,51]. Furthermore, the GM has other metabolic functions such as bile acid transformation by microbial enzymes for cholesterol and glucose metabolism, amino acid synthesis and vitamin production [52,53]. Another beneficial function for the host is short-chain fatty acid (SCFAs) production, which includes acetate, butyrate, and propionate required for energy production and cholesterol synthesis [54,55]. As ME/CFS is a systemic disease, GI disturbances are another class of symptoms commonly reported [56,57,58]. Indeed, comorbidities such as irritable bowel syndrome (IBS) or Crohn′s disease may be found in ME/CFS individuals, thus suggesting a possible role of the gut microbiome in disease progression [59,60]. However, whether and how the GM is involved in ME/CFS pathogenesis and development is still unknown. Here, we briefly review the most relevant studies addressing how dysbiosis and intestinal permeability may contribute to disease phenotype, and we discuss the possible therapeutic applications aimed at restoring eubiosis and intestinal barrier integrity in the context of ME/CFS. 

## 2. Main Findings 

### 2.1. Alterations of Human Microbiome in ME/CFS

**Figure 1:**
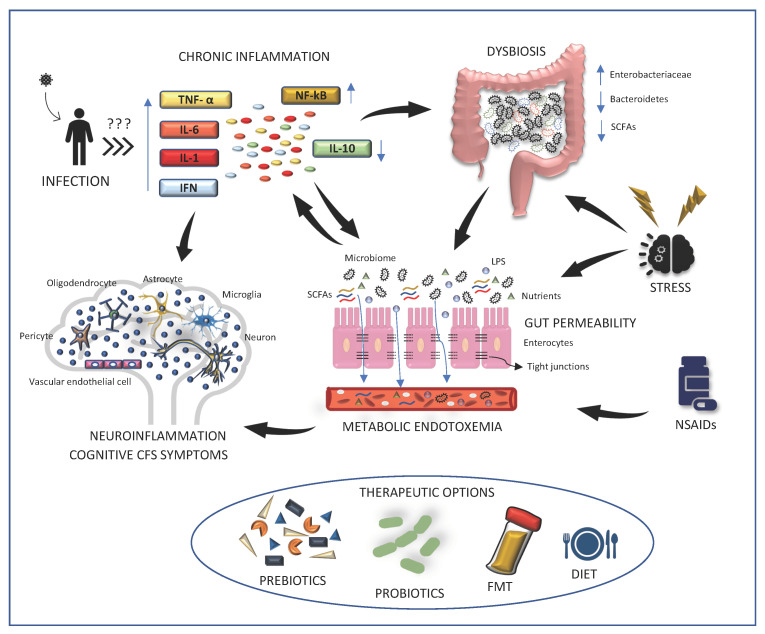
Role of dysbiosis and gut permeability in ME/CFS pathogenesis.

In the past years, studies have been conducted to investigate the kind of alterations taking place in the gut microbiome in ME/CFS and their implications for those suffering from ME/CFS. Significant dysregulations in the overall composition of microbiota and shifted ratios between several bacterial taxa in comparison to healthy controls have been detected ([61,62], Figure 1, Table 1). For example, a modified microbiome was found in saliva, gut, and feces of ME/CFS cases, linking the GM to the disease [12,63]. Moreover, when 16S ribosomal ribonucleic acid (rRNA) sequencing was used to compare stool samples from 43 ME/CFS individuals and 36 healthy controls, an altered GM composition and imbalance in microbial diversity have been reported ([64] Table 1). Subsequently, similar results were obtained using the same technique [13,14,63,65,66]. Interestingly, a striking decrease in relative abundance and diversity of *Firmicutes* bacteria, and a higher number of *Bacteroidetes* was detected [14]. Often, a lower *Bacteroides*/*Firmicutes* ratio can be accompanied by an increase in *Enterobacteriaceae*, therefore suggesting a complete reshuffling of the gut microbiota composition [63,64]. Since shifts in microbial ratios have also been identified in autoimmune conditions such as Crohn′s disease, Systemic Lupus Erythematosus 2, and Diabetes Type 2, it would be interesting to investigate whether the microbiome may be linked to ME/CFS autoimmune manifestations, if they occur [63,67,68,69,70]. While environmental and genetic factors can alter the microbiome [42,44], changes in GM composition according to geographical origin should also be considered in ME/CFS [64]. In this respect, studies involving matched healthy controls are crucial. When accounting for these differences, Nagy-Szakal et al. report a differential microbiota composition in ME/CFS cases with or without IBS comorbidity when compared to the same number of matched controls. Indeed, while an increase in *Alipstes* and a decrease in *Faecalibacterium* seem to characterize ME/CFS individuals who also present IBS, a rise in unclassified *Bacteroides*, but not in *Bacteroides vulgatus*, appears typical of ME/CFS without IBS comorbidity [13]. However, as disturbances may arise due to the high prevalence of IBS comorbidity in individuals with ME/CFS, these results should be confirmed in larger cohorts before drawing any conclusion [13].

GM dysbiosis may also represent a cause of increased gut permeability [60]. In this respect, a correlation between changes in GM and a higher level of inflammation was observed in some studies [60,64]. Moreover, increased commensal bacterial translocation and enhanced gut inflammation have been found in ME/CFS cases compared to controls, as discussed in more detail in Section 2.2 [60,74,75] (Figure 1). Although the exact mechanism behind this phenomenon largely remains unknown, one hypothesis is that the rise in *Enterobacteriaceae* found in dysbiosis may mediate intestinal inflammation and permeability, as increased levels of lipopolysaccharide derived from these bacteria is detected in ME/CFS [74,76,77] (Figure 1). However, it should be noted that this is far from being proven, and more research is needed to address this point. Another possibility is that bacterial metabolites contribute to the disease by interfering with the estrogen receptor and Vitamin D receptor pathways, as the latter is also involved in development of autoimmune disorders, which often occur as comorbidities of ME/CFS as mentioned previously, but this topic remains to be addressed [64,78,79]. Last, when searching for a possible mechanism for how dysbiosis influences ME/CFS pathogenesis, the gut-brain-axis, and the autonomic and enteric nervous systems should also be considered [60,80].

Although the importance of gut microbiome in health and disease is becoming more and more prominent, several limitations still need to be addressed in respect to ME/CFS. Indeed, if the data cited above report evidence for a dysregulated gut microbiota composition, it is also true that contradictory studies are present in the literature. For example, when 18S rRNA sequencing was used to analyze eukaryotic diversity in ME/CFS cases compared to controls, insignificant differences were reported [61]. Likewise, even though alterations in the human gut microbiome (i.e., the multitude of genes of the gut microbiota), have been observed in multiple studies in ME/CFS cases, results have failed to be reproduced between studies, likely due to study design [12,14,81]. The reason for this discrepancy could be found, at least in part, in the narrowness of the cohort analyzed in each study. In this respect, in order to have reliable and statistically significant results new investigations should be carried out involving more participants, both ME/CFS cases and controls. Similarly, the idea of using rRNA sequencing as a new diagnostic tool in ME/CFS, although attractive, has yet to be validated to avoid misdiagnosis. Altogether, these data point out that gut microbiota alterations seem to characterize ME/CFS in those affected, but the role of dysbiosis in disease pathogenesis and progression should be further investigated.

### 2.2. Increased Gut Permeability in ME/CFS

The intestinal barrier is a single-cell epithelial layer that allows the selective absorption of nutrients, electrolytes, and water through a mucous membrane. In health, epithelial cells are tightly connected by desmosomes, adherens junctions and tight junctions, which are made up of occludin, claudins, and junctional adhesion molecules respectively. Thus, intraluminal translocation of bacteria and toxins into the bloodstream is prevented [82]. However, when homeostasis is altered, for example due to gut inflammation, dysbiosis, chronic NSAID intake, or stress, the barrier integrity is lost and commensal bacteria can reach the bloodstream (Figure 1) [60,82,83]. The presence of circulating lipopolysaccharide (LPS) derived from gram-negative endobacteria, also known as metabolic endotoxemia, then activates the inflammatory TLR4 pathway and immune cells produce pro-inflammatory cytokines and LPS-directed IgM/IgA, thus enhancing systemic inflammation [74,76,84,85].

Metabolic endotoxemia and gut permeability have already been considered in the pathophysiological mechanism of several diseases such as obesity, diabetes, nonalcoholic fatty liver disease, atherosclerosis, metabolic syndrome, or septic shock, as well as ME/CFS [60,75,84,85,86]. In this respect, serum IgA and IgM levels against LPS of enterobacteria are significantly higher in ME/CFS cases than controls, and correlate with disease severity [74]. Likewise, raised IgA response to commensal bacteria and enhanced inflammation have been reported in 128 ME/CFS cases when compared to healthy volunteers [76]. Remarkably, significant improvement was obtained if a leaky gut diet was combined with anti-inflammatory and anti-oxidative substances, thus suggesting a new therapeutic approach in ME/CFS treatment [77]. Similar results were also obtained in depressed patients, suggesting that gut permeability and consequently enhanced immune response might explain overlap between major depressive disorder (MDD) and ME/CFS cognitive symptom [87,88]. A growing body of evidence demonstrates the importance of neuroinflammation in the development of neurodegenerative and neuroprogressive diseases [89,90]. Given the ability of bacterial translocation to drive systemic inflammation, blood-brain barrier disruption and neuroinflammation, some authors hypothesize that this mechanism might explain the onset of neurological abnormalities in ME/CFS, but this remains to be proven [83,91,92]. Based on this hypothesis, leaky gut targeting may reduce both gastrointestinal and cognitive symptoms, thus representing a promising approach in ME/CFS therapy but more research is needed before drawing conclusions.

There is evidence that ME/CFS could be classified as an autoimmune disease [93], and gut permeability may also play a role in this context. After a viral trigger, dysbiosis and genetic predisposition favor the generation of immune cell clones prone to autoreactivity, leading to self-antigen immunization and autoimmunity [16]. In addition, a link between fatigue, autoimmunity, and intestinal barrier breakdown has also been established [94]. The fact that dysbiosis and bacterial translocation cause an increase in pro-inflammatory cytokines (i.e., IL-1 and TNF-α) is an additional mechanism that could explain the relationship between gut, ME/CFS and autoimmunity [95]. However, the role and the importance of autoimmunity in ME/CFS pathophysiology are not yet clear, and more studies are needed to confirm these suggestions.

A complex relationship between dysbiosis, intestinal permeability, chronic inflammation, and cognitive symptoms is reported in ME/CFS. A viral infection may represent an important trigger of systemic inflammation, which in turn promotes dysbiosis and neuroinflammation. In these conditions, *Enterobacteriaceae* growth is favored, while *Bacteroidetes* and SCFA production are impaired. This imbalanced gut composition, together with chronic inflammation, stress and NSAIDs prolonged intake, favors tight-junction disruption and leaky gut. While in base-line conditions only nutrients and SCFAs can reach the bloodstream, upon intestinal barrier integrity loss, bacterial and LPS translocation are also possible. Given that the resulting metabolic endotoxemia exacerbates pro-inflammatory cytokine production and release, this chronic-low grade inflammation contributes to neuroinflammation and neurological abnormalities.

Therapeutic options aimed at restoring gut barrier integrity and eubiosis have been proposed. Among those, prebiotics, probiotics, fecal microbiota transplantation (FMT), and diet interventions have all shown promising results, but more studies are needed to determine their efficacy.

### 2.3. Oxidative Stress and Inflammation in Disease Pathogenesis

Oxidative stress refers to a condition in which high levels of intracellular reactive oxygen species (ROS) accumulate and cause protein, lipid, and DNA damage [96]. Although antioxidants are supposed to counteract the buildup of ROS, their levels in chronic conditions, such as IBD, remain low [97]. In addition, chronic low-grade inflammation and oxidative stress are both associated with ME/CFS [60,98]. For example, an increase in oxidative stress level and a decrease in antioxidant levels in resting conditions have been reported in ME/CFS cases when compared to controls [99]. Moreover, elevated urinary 8-hydroxy-deoxoguanosine (8-OHdG) levels, a well-known marker of oxidative DNA damage, was shown to correlate with malaise and depression in ME/CFS [100]. Similar to IBS, high levels of pro-inflammatory cytokines (i.e., IFN-γ, IL-4, IL-5, TGF-α and IL-1) are also detected in ME/CFS [101,102]. Although it is not yet clear whether inflammation can directly cause fatigue, the enhancement of 92 circulating inflammatory markers in ME/CFS individuals resembles the analysis obtained for Q fever fatigue [103]. Given the lack of defined biomarkers in ME/CFS, the possibility of relying on inflammatory, oxidative/nitrosative stress, and antioxidants markers has been proposed [60,99,104].

Although several factors contribute to the establishment of inflammation and oxidant/antioxidant imbalance (i.e., viral infection, reduced antioxidants, stress, depression [60,75,105]), dysbiosis, and metabolic endotoxemia also play an important role [60,83,91]. In this respect, a model has been established according to which stress, dysbiosis, and systemic inflammation all contribute to reducing the tight-junction protein occludin, thus causing the intestinal lining to lose its barrier function [60,82,83]. Increased gut permeability, in turn, further exacerbates chronic inflammation via endotoxemia and TLR4 pathway activation, leading to neuroinflammation and oxidative/nitrosative stress [83,85]. As evident in Idiopathic Chronic Fatigue (ICF), oxidative stress may finally represent a key pathophysiological mechanism in ME/CFS [83,106,107]. Even though it still requires fundamental validations, if this model turns out to be true, it will certainly constitute a new key target in ME/CFS treatment, thus confirming the central role of gut homeostasis in both gastrointestinal and extra-intestinal disease pathogenesis.

### 2.4. Therapies Aimed at Microbiota May Alleviate ME/CFS Symptoms

Given the frequent association of ME/CFS with chronic inflammation, dysbiosis and gut permeability [108], it is worth speculating that approaches aimed at replenishing the microbial balance, restoring mucosal barrier integrity, and lowering inflammation may be therapeutically relevant. Prebiotics, probiotics, specific diet, particular molecule intake, and fecal transplantation have been proposed, in this respect (Figure 1) [109]. NADH, probiotics, high cocoa polyphenol rich chocolate and Coenzyme Q10 proved all capable of improving fatigue in ME/CFS-diagnosed cases, but questions remain on whether the results can be replicated on a larger sample size [110].

#### 2.4.1. Probiotics

*Probiotics* are living microorganisms which normally reside in the human body. *Lactobacilli* spp., *E. coli*-Nisle 1917, *Bifodobacteria* spp., some *Streptococcus* types, and the yeast *Saccharomyces boulardii* are all considered probiotics [60]. Recently, their application as adjuvant therapy in IBS treatment mostly showed positive results [111,112,113,114,115,116,117,118,119,120,121,122,123,124]. In addition, administration of *Akkermansia muciniphila* and *Lactobacillus sakei* OK67 to high-fat diet (HFD) fed mice were independently able to enhance tight-junction function, increasing occludin gene expression and decreasing intestinal permeability [125,126]. Remarkably, during L. sakei OK67 treatment, a significant decrease in the inflammatory markers TNF-α, IL-1β and NF-κB has also been reported [126]. In the context of ME/CFS, the same promising results were replicated applying an 8-week long treatment of four probiotic mixtures [127]. Moreover, the administration of *Bifidobacterium infantis* 35624 to 48 ME/CFS cases confirmed the ability of probiotics to reduce the systemic pro-inflammatory markers CRP, TNF-α and IL-6 [128].

Anxiety, depression, and psychiatric disorders are often found in ME/CFS affected individuals [129] and finding an alternative to the currently employed psychotropic medications is crucial. Results from a 12-week randomized, double-blind, and placebo controlled clinical trial report that a mixture of *Lactobacillus helveticus* R0052 and *Bifidobacterium longum* R0175 could be effective in decreasing inflammation and improving psychiatric manifestations in MDD patients following a gluten-free diet [130]. Since both MDD and ME/CFS show psychiatric symptom overlap [131], it is interesting to see whether probiotic use in chronic fatigue would prove equally beneficial. Preliminary evidence suggests that a significant drop in anxiety, associated with eubiosis reestablishment, can be observed if *Lactobacillus casei* strain Shirota is administered daily for 2 months in ME/CFS cases [132]. In addition, improvements in neurocognitive functions among *L. paracasei* spp. *paracasei* F19, *L. acidophilus* NCFB 1748 and *B. lactis* Bb12 receiving ME/CFS-diagnosed individuals are particularly notable [109]. Overall, these studies show that probiotics, alone or in combination, will probably emerge as a remedy supporting ME/CFS therapy.

#### 2.4.2. Prebiotics

Prebiotics are non-digestible carbohydrate nutrients which are used as food by the GM. Fructo-oligosaccharides and galacto-oligosaccharides are the two main prebiotic classification groups [133]. Upon bacterial degradation, they produce SCFAs that diffuse via systemic circulation, hence influencing both gastrointestinal and extra-intestinal functionality [134]. Given their ability to selectively promote the expansion of only some intestinal microorganisms and revising gut microbiota makeup and function [133], they are proposed as promising adjuvant therapy in many diseases (e.g., IBS, Crohn′s disease, bowel motility, autism, obesity and colorectal cancer) [133]. Multiple oligosaccharides have proven effective in reversing microbiota dysbiosis through *Lactobacilli* growth promotion, *Proteobacteria* reduction, and *Firmicutes/Bacteroidetes* ratio decrease in diet-induced obese rats and mice [135,136,137]. In addition, significant amelioration of gut permeability and systemic inflammation have also been reported. Rats and mice fed with prebiotics, such as bovine milk oligosaccharides, oligofructose-enriched inulin, spirulina platensis, and FOS/GOS, showed lower plasma LPS, decrease in serum pro-inflammatory cytokine levels, reduced gut inflammation and improved tight-junction integrity [135,136,137,138,139]. Altogether, these studies suggest that prebiotics may be helpful for ME/CFS cases presenting dysbiosis, leaky gut and systemic basal inflammation, but clinical trials are needed before drawing further conclusions.

#### 2.4.3. Diet

A change in dietary habit is a rapid, reproducible and direct way of modifying the gut microbiota [140]. Diet, other than being involved in some disease pathophysiology, if adequate and taken at set times, is capable of balancing microbiota composition and mitigating inflammation, similar to prebiotics [141,142]. In the last few years, IBS, obesity, and Crohn′s patients have benefited from this therapy, and dietary interventions have also been considered in the neuropsychiatric field [143,144,145,146,147,148,149].

Glucose/fructose-based diets and long-term protein-based diets have been correlated with dysbiosis, leaky gut, increased systemic inflammation and increased levels of plasma endotoxins [150,151]. Consequently, gluten-free diets, starch and sucrose-reduced diet, and dietary regimens aimed at lowering caloric intake can decrease C-reactive protein (CRP) and LPS binding protein levels, counteract intestinal permeability, and ameliorate gastrointestinal and extra-intestinal symptoms of IBS and obesity [130,152,153]. Similarly, microbiota diversity and metabolic endotoxemia are improved by polyunsaturated fatty acid omega-3 intake, and polyphenol and fiber consumption are preferred [142,154]. Eicosapentaenoic acid which is found in omega-3 rich fish oil has also been found to alleviate symptoms in ME/CFS cases [155,156]. In diet-induced obese rats and mice, some benefits can also be achieved by specific nutrient integration. In this respect, apple polysaccharides, flos lanicera administration and Bofutsushasan (a Japanese herbal medicine) have proven effective in favoring *Lactobacillus* and *Bacteroidetes* growth, enhancing tight junction function and reducing the pro-inflammatory cytokines TNF-α and IL-6 [157,158,159]. Additionally, integration of *Sarcodon imbricatus* or intake of a mixture composed of *Angelica gigas*, *Cnidium officinale*, and *Paeonia lactiflora*, proved effective in restoring the oxidant/antioxidant homeostasis and in reducing fatigue in ME/CFS mouse models [160,161].

Although more clinical trials are needed in humans, these results indicate that the ability to act on microbiome makeup, gut permeability, inflammation and neurocognitive symptoms at the same time proposes dietary intervention as a promising additional adjuvant approach in ME/CFS treatment.

#### 2.4.4. Fecal Microbiota Transplantation (FMT)

Fecal microbiota transplantation (FMT), also known as stool transplantation or bacteriotherapy, is the process of transplanting stool from a healthy donor into a patient′s intestine [162]. The aim of the therapy is to restore dysbiosis by infusing a balanced and healthy microbiota population into the gut of the recipient. In most cases the transplantation takes place via colonoscopy, but enema or orally administered capsules are also available [163,164]. Although it is only approved for recurrent or refractory *Clostridium difficile* infection treatment [165], FMT is now being tested as an experimental therapeutic option for primary *Clostridium difficile* infection, obesity, insulin resistance, metabolic syndrome, metabolic fatty acid liver disease, fibromyalgia, ulcerative colitis, Crohn′s disease, ME/CFS, functional constipation, IBS, and even cancer [164,166,167,168,169]. In addition, several neuropsychiatric disorders have been proposed as potentially benefitting from FMT. Studies are being carried out using stool transplantation in autism, Parkinson′s Disease, Alzheimer′s Disease, and Multiple Sclerosis, but the success of these trials is debatable [170,171,172]. Recently, promising perspectives came from the use of FMT in immune-checkpoint inhibitor-associated colitis, IBS, and IBD, but larger cohort trials are needed [162,164,173]. FMT ability to decrease inflammation, reduce intestinal permeability via SCFA production and restore immune dysbiosis [174] proposes this nascent therapy as a promising approach also in ME/CFS treatment. In a study of 34 ME/CFS participants who received FMT, 41% showed persistent relief after 11–28 months, while 35% reported only little or late relief [175]. Moreover, a 70% response rate was obtained when 13 non-pathogenic bacteria were administered via colonoscopy in 60 ME/CFS individuals. Additionally, at 15–20 years follow up, 58% of cases reported maintained response without recurrence [176].

Despite the potential of FMT in a wide range of diseases, limitations are still evident. Lack of consistency and shared standard protocols, selection criteria, route of administration, therapy duration, long-term risks, and donor selection are all open questions that have not yet been addressed [162,167,170,177,178,179]. Moreover, several authors underline that no solid conclusions can be drawn from existing studies, and larger clinical trials are needed in order to clarify FMT efficiency in various human disorders [162,170,174,180,181]. It would also be worthwhile to see if the multiple donor FMT proved more effective than the single donor approach, as already suggested in the literature [182].

While several limitations exist, these data indicate that FMT application in multiple intestinal dysbiosis-associated extra-intestinal diseases may soon represent a novel therapeutic approach for ME/CFS cases.

## 3. Discussion

Altogether, this short review summarizes the main findings concerning dysbiosis and gut permeability in ME/CFS. While GM homeostasis has proved to be fundamental in many diseases, its role in ME/CFS pathogenesis and disease development is still partially unclear and needs to be fully addressed to enable proper treatment of the disease. Studies on larger cohorts, use of consistent criteria for the diagnosis of ME/CFS, and reduction of confounding variables by controlling factors that influence microbiome composition prior to sample collection are needed in this respect. At the same time, therapeutic applications aimed at eubiosis re-establishment and leaky-gut prevention should be tested further in humans, as current promising insights are often based on data from mice and rats. Similarly, microbiome alterations or metabolic endotoxemia should be considered as potential disease biomarkers, even though GI symptoms overlap with those of other disorders and may represent a concern for precise differential diagnosis. Nevertheless, the importance of the GM in ME/CFS is evident through the links between GM alterations, inflammation, autoimmunity, and the gut-brain axis. Overall, we give an overview of the promising microbiome-based therapeutic applications for the chronic and strongly debilitating disease that is ME/CFS, and encourage deeper research in this field.

## Figures and Tables

**Table 1:** Summary of studies concerning dysbiosis in ME/CFS.

Reference	Journal	Participants	Classification Criteria	Analysis Performed	Results
Giloteaux et al., 2016 [71]	Am Jour Case Rep	A pair of 34 year old monozygotic male twins, 1 ME/CFS and 1 control	Fukuda (1994) [4]	Two-day CPET; stool biochemical and molecular analysis; 16S RNA sequencing	**↓** Microbial diversity **↓** *Faecalibacterium* and *Bifidobacterium*
Shukla et al., 2015 [66]	PLOS One	10 ME/CFS and 10 matched healthy controls	Fukuda (1994) [4]	Maximal exercise challenge, stool examination before and 15 min, 48 h, 72 h after exercise. PCR and 16S rRNA sequence	**↑** Abundance changes of major bacterial phyla (after exercise) **↓** Bacterial clearance (after exercise)
Kitami et al., 2020 [65]	Sci Rep	48 ME/CFS and 52 controls	Fukuda (1994) [4] and International Consensus Criteria (2011) [5]	Stool microbiome analysis by DNA extraction and 16S rRNA sequencing	**↑** *Coprobacillus*, *Eggerthella* and *Blautia*
Mandarano et al., 2018 [61]	PeerJ	49 ME/CFS and 39 healthy controls	Fukuda (2004) [4]	18S rRNA sequencing in stool samples	**↓** Eukaryotic diversity (nonsignificant) **↑** *Basidiomycota*/*Ascomycota* ratio (nonsignificant)
Nagy-Szakal et al., 2017 [13]	Microbiome	50 ME/CFS and 50 matched healthy controls	Fukuda (2004) [4] and/or Canadian Criteria (2003) [3]	Fecal bacterial metagenomics (shotgun metagenomic sequences)	**↑** Dysbiosis **↑** Alistipes (in ME/CFS with IBS), *Bacteroides* (in ME/CFS without IBS) **↓** *Faecalibacterium* (in ME/CFS with IBS), *Bacteroides* vulgatus (in ME/CFS without IBS)
Lupo et al., 2021 [63]	Sci Rep	35 ME/CFS and 70 healthy controls (35 had relatives with ME/CFS and 35 not)	Fukuda (2004) [4]	Fecal bacterial analysis by 16S rRNA Illumina sequencing	**↓** *Anaerostipes* (*Lachnospiraceae*) **↑** *Bacteroides* and *Phascolarctobacterium*
Giloteaux et al., 2016 [14]	Microbiome	49 ME/CFS and 39 healthy controls	Fukuda (2004) [4]	16S rRNA sequencing from stool	**↓** Diversity **↓** *Firmicutes* phylum **↑** Pro-inflammatory species (*Proteobacteria* species)
Frémont et al., 2013 [64]	Anaerobe	43 ME/CFS and 36 healthy controls	Fukuda (1994) [4]	High-throughput 16S rRNA sequencing from stool samples	**↑** Lactonifactor and Alistipes **↓** Several *Firmicutes* populations
Sheedy et al., 2009 [62]	In Vivo	108 ME/CFS and 177 healthy controls	Holmes (1988) [72]/Fukuda (1994) [4]/Canadian Definition Criteria (2003) [3]	Fecal sample collection and identification of facultative anaerobic organisms using standard criteria [73]	**↑** Dlactic acid producing *Enterococcus* and *Streptococcus* spp.

CPET: cardiopulmonary exercise test; **↓** decrease; **↑** increase.

## Data Availability

Data sharing not applicable.

## References

[1] Prins J.B., van der Meer J.W., Bleijenberg G. (2006). Chronic Fatigue Syndrome. Lancet.

[2] Sharpe M.C., Archard L.C., Banatvala J.E., Borysiewicz L.K., Clare A.W., David A., Edwards R.H., Hawton K.E., Lambert H.P., Lane R.J. (1991). A Report—Chronic Fatigue Syndrome: Guidelines for Research. J. R. Soc. Med..

[3] Carruthers B., Jain A., de Meirleir K., Peterson D., Klimas N., Lerner A., Bested A., Pierre F., Joshi P., Powles A. (2003). Myalgic Encephalomyelitis/Chronic Fatigue Syndrome: Clinical Working Case Definition, Diagnostic and Treatment Protocols. J. Chronic Fatigue Syndr..

[4] Fukuda K. (1994). The Chronic Fatigue Syndrome: A Comprehensive Approach to Its Definition and Study. Ann. Intern. Med..

[5] Carruthers B.M., van de Sande M.I., de Meirleir K.L., Klimas N.G., Broderick G., Mitchell T., Staines D., Powles A.C.P., Speight N., Vallings R. (2011). Myalgic Encephalomyelitis: International Consensus Criteria. J. Intern. Med..

[6] Bested A., Marshall L. (2015). Review of Myalgic Encephalomyelitis/Chronic Fatigue Syndrome: An Evidence-Based Approach to Diagnosis and Management by Clinicians. Rev. Environ. Health.

[7] Mandarano A.H., Maya J., Giloteaux L., Peterson D.L., Maynard M., Gottschalk C.G., Hanson M.R. (2020). Myalgic Encephalomyelitis/Chronic Fatigue Syndrome Patients Exhibit Altered T Cell Metabolism and Cytokine Associations. J. Clin. Investig..

[8] Lorusso L., Mikhaylova S.V., Capelli E., Ferrari D., Ngonga G.K., Ricevuti G. (2009). Immunological Aspects of Chronic Fatigue Syndrome. Autoimmun. Rev..

[9] Wirth K., Scheibenbogen C. (2020). A Unifying Hypothesis of the Pathophysiology of Myalgic Encephalomyelitis/Chronic Fatigue Syndrome (ME/CFS): Recognitions from the Finding of Autoantibodies against SS2-Adrenergic Receptors. Autoimmun. Rev..

[10] Maes M., Twisk F.N.M., Kubera M., Ringel K. (2012). Evidence for Inflammation and Activation of Cell-Mediated Immunity in Myalgic Encephalomyelitis/Chronic Fatigue Syndrome (ME/CFS): Increased Interleukin-1, Tumor Necrosis Factor-α, PMN-Elastase, Lysozyme and Neopterin. J. Affect. Disord..

[11] Cortes Rivera M., Mastronardi C., Silva-Aldana C., Arcos-Burgos M., Lidbury B. (2019). Myalgic Encephalomyelitis/Chronic Fatigue Syndrome: A Comprehensive Review. Diagnostics.

[12] Magnus P., Gunnes N., Tveito K., Bakken I.J., Ghaderi S., Stoltenberg C., Hornig M., Lipkin W.I., Trogstad L., Håberg S.E. (2015). Chronic Fatigue Syndrome/Myalgic Encephalomyelitis (CFS/ME) Is Associated with Pandemic Influenza Infection, but Not with an Adjuvanted Pandemic Influenza Vaccine. Vaccine.

[13] Nagy-Szakal D., Williams B.L., Mishra N., Che X., Lee B., Bateman L., Klimas N.G., Komaroff A.L., Levine S., Montoya J.G. (2017). Fecal Metagenomic Profiles in Subgroups of Patients with Myalgic Encephalomyelitis/Chronic Fatigue Syndrome. Microbiome.

[14] Giloteaux L., Goodrich J.K., Walters W.A., Levine S.M., Ley R.E., Hanson M.R. (2016). Reduced Diversity and Altered Composition of the Gut Microbiome in Individuals with Myalgic Encephalomyelitis/Chronic Fatigue Syndrome. Microbiome.

[15] Navaneetharaja N., Griffiths V., Wileman T., Carding S. (2016). A Role for the Intestinal Microbiota and Virome in Myalgic Encephalomyelitis/Chronic Fatigue Syndrome (ME/CFS)?. J. Clin. Med..

[16] Blomberg J., Gottfries C.-G., Elfaitouri A., Rizwan M., Rosén A. (2018). Infection Elicited Autoimmunity and Myalgic Encephalomyelitis/Chronic Fatigue Syndrome: An Explanatory Model. Front. Immunol..

[17] Sullivan P.F., Evengard B., Jacks A., Pedersen N.L. (2005). Twin Analyses of Chronic Fatigue in a Swedish National Sample. Psychol. Med..

[18] Glassford J.A.G. (2017). The Neuroinflammatory Etiopathology of Myalgic Encephalomyelitis/Chronic Fatigue Syndrome (ME/CFS). Front. Physiol..

[19] Hickie I., Bennett B., Lloyd A., Heath A., Martin N. (1999). Complex Genetic and Environmental Relationships between Psychological Distress, Fatigue and Immune Functioning: A Twin Study. Psychol. Med..

[20] Van de Putte E., van Doornen L., Engelbert R., Kuis W., Kimpen J., Uiterwaal C. (2006). Mirrored Symptoms in Mother and Child with Chronic Fatigue Syndrome. Pediatrics.

[21] Albright F., Light K., Light A., Bateman L., Cannon-Albright L.A. (2011). Evidence for a Heritable Predisposition to Chronic Fatigue Syndrome. BMC Neurol..

[22] Dibble J.J., McGrath S.J., Ponting C.P. (2020). Genetic Risk Factors of ME/CFS: A Critical Review. Hum. Mol. Genet..

[23] Jacobson S.K., Daly J.S., Thorne G.M., McIntosh K. (1997). Chronic Parvovirus B19 Infection Resulting in Chronic Fatigue Syndrome: Case History and Review. Clin. Infect. Dis..

[24] Aoki R., Kobayashi N., Suzuki G., Kuratsune H., Shimada K., Oka N., Takahashi M., Yamadera W., Iwashita M., Tokuno S. (2016). Human Herpesvirus 6 and 7 Are Biomarkers for Fatigue, Which Distinguish between Physiological Fatigue and Pathological Fatigue. Biochem. Biophys. Res. Commun..

[25] Niller H.H., Wolf H., Ay E., Minarovits J. (2011). Epigenetic Dysregulation of Epstein-Barr Virus Latency and Development of Autoimmune Disease. Adv. Exp. Med. Biol..

[26] Kerr J.R. (2016). The Role of Parvovirus B19 in the Pathogenesis of Autoimmunity and Autoimmune Disease. J. Clin. Pathol..

[27] Kerr J.R., Bracewell J., Laing I., Mattey D.L., Bernstein R.M., Bruce I.N., Tyrrell D.A.J. (2002). Chronic Fatigue Syndrome and Arthralgia Following Parvovirus B19 Infection. J. Rheumatol..

[28] Seishima M., Mizutani Y., Shibuya Y., Arakawa C. (2008). Chronic Fatigue Syndrome after Human Parvovirus B19 Infection without Persistent Viremia. Dermatology.

[29] Cameron B., Flamand L., Juwana H., Middeldorp J., Naing Z., Rawlinson W., Ablashi D., Lloyd A. (2010). Serological and Virological Investigation of the Role of the Herpesviruses EBV, CMV and HHV-6 in Post-Infective Fatigue Syndrome. J. Med. Virol..

[30] Rasa S., Nora-Krukle Z., Henning N., Eliassen E., Shikova E., Harrer T., Scheibenbogen C., Murovska M., Prusty B.K. (2018). Chronic Viral Infections in Myalgic Encephalomyelitis/Chronic Fatigue Syndrome (ME/CFS). J. Transl. Med..

[31] Soto N.E., Straus S.E. (2000). Chronic Fatigue Syndrome and Herpesviruses: The Fading Evidence. Herpes J. IHMF.

[32] Levine P.H., Jacobson S., Pocinki A.G., Cheney P., Peterson D., Connelly R.R., Weil R., Robinson S.M., Ablashi D.V., Salahuddin S.Z. (1992). Clinical, Epidemiologic, and Virologic Studies in Four Clusters of the Chronic Fatigue Syndrome. Arch. Intern. Med..

[33] Blomberg J., Rizwan M., Böhlin-Wiener A., Elfaitouri A., Julin P., Zachrisson O., Rosén A., Gottfries C.-G. (2019). Antibodies to Human Herpesviruses in Myalgic Encephalomyelitis/Chronic Fatigue Syndrome Patients. Front. Immunol..

[34] Burbelo P.D., Bayat A., Wagner J., Nutman T.B., Baraniuk J.N., Iadarola M.J. (2012). No Serological Evidence for a Role of HHV-6 Infection in Chronic Fatigue Syndrome. Am. J. Transl. Res..

[35] Domingues T.D., Grabowska A.D., Lee J.-S., Ameijeiras-Alonso J., Westermeier F., Scheibenbogen C., Cliff J.M., Nacul L., Lacerda E.M., Mouriño H. (2021). Herpesviruses Serology Distinguishes Different Subgroups of Patients From the United Kingdom Myalgic Encephalomyelitis/Chronic Fatigue Syndrome Biobank. Front. Med..

[36] Clauw D.J. (2010). Perspectives on Fatigue from the Study of Chronic Fatigue Syndrome and Related Conditions. PM&R.

[37] Simani L., Ramezani M., Darazam I.A., Sagharichi M., Aalipour M.A., Ghorbani F., Pakdaman H. (2021). Prevalence and Correlates of Chronic Fatigue Syndrome and Post-Traumatic Stress Disorder after the Outbreak of the COVID-19. J. Neurovirol..

[38] Kennedy G., Khan F., Hill A., Underwood C., Belch J.J.F. (2010). Biochemical and Vascular Aspects of Pediatric Chronic Fatigue Syndrome. Arch. Pediatr. Adolesc. Med..

[39] Tilg H., Adolph T.E., Gerner R.R., Moschen A.R. (2018). The Intestinal Microbiota in Colorectal Cancer. Cancer Cell.

[40] Ogino S., Nowak J.A., Hamada T., Phipps A.I., Peters U., Milner D.A., Giovannucci E.L., Nishihara R., Giannakis M., Garrett W.S. (2018). Integrative Analysis of Exogenous, Endogenous, Tumour and Immune Factors for Precision Medicine. Gut.

[41] Rinninella E., Raoul P., Cintoni M., Franceschi F., Miggiano G., Gasbarrini A., Mele M. (2019). What Is the Healthy Gut Microbiota Composition? A Changing Ecosystem across Age, Environment, Diet, and Diseases. Microorganisms.

[42] Bäckhed F., Ley R.E., Sonnenburg J.L., Peterson D.A., Gordon J.I. (2005). Host-Bacterial Mutualism in the Human Intestine. Science.

[43] Thursby E., Juge N. (2017). Introduction to the Human Gut Microbiota. Biochem. J..

[44] Nicholson J.K., Holmes E., Kinross J., Burcelin R., Gibson G., Jia W., Pettersson S. (2012). Host-Gut Microbiota Metabolic Interactions. Science.

[45] Prakash S., Rodes L., Coussa-Charley M., Tomaro-Duchesneau C., Tomaro-Duchesneau C., Coussa-Charley M. (2011). Rodes Gut Microbiota: Next Frontier in Understanding Human Health and Development of Biotherapeutics. Biol. Targets Ther..

[46] Morais L.H., Schreiber H.L., Mazmanian S.K. (2021). The Gut Microbiota–Brain Axis in Behaviour and Brain Disorders. Nat. Rev. Microbiol..

[47] Mayer E.A., Tillisch K., Gupta A. (2015). Gut/Brain Axis and the Microbiota. J. Clin. Investig..

[48] Atarashi K., Tanoue T., Shima T., Imaoka A., Kuwahara T., Momose Y., Cheng G., Yamasaki S., Saito T., Ohba Y. (2011). Induction of Colonic Regulatory T Cells by Indigenous Clostridium Species. Science.

[49] Ivanov I.I., Atarashi K., Manel N., Brodie E.L., Shima T., Karaoz U., Wei D., Goldfarb K.C., Santee C.A., Lynch S.V. (2009). Induction of Intestinal Th17 Cells by Segmented Filamentous Bacteria. Cell.

[50] Khan R., Petersen F.C., Shekhar S. (2019). Commensal Bacteria: An Emerging Player in Defense against Respiratory Pathogens. Front. Immunol..

[51] Zhang H., DiBaise J.K., Zuccolo A., Kudrna D., Braidotti M., Yu Y., Parameswaran P., Crowell M.D., Wing R., Rittmann B.E. (2009). Human Gut Microbiota in Obesity and after Gastric Bypass. Proc. Natl. Acad. Sci. USA.

[52] Malaguarnera L. (2020). Vitamin D and Microbiota: Two Sides of the Same Coin in the Immunomodulatory Aspects. Int. Immunopharmacol..

[53] Wahlström A., Sayin S.I., Marschall H.-U., Bäckhed F. (2016). Intestinal Crosstalk between Bile Acids and Microbiota and Its Impact on Host Metabolism. Cell Metab..

[54] Canfora E.E., Jocken J.W., Blaak E.E. (2015). Short-Chain Fatty Acids in Control of Body Weight and Insulin Sensitivity. Nat. Rev. Endocrinol..

[55] Morrison D.J., Preston T. (2016). Formation of Short Chain Fatty Acids by the Gut Microbiota and Their Impact on Human Metabolism. Gut Microbes.

[56] Johnston S., Staines D., Marshall-Gradisnik S. (2016). Epidemiological Characteristics of Chronic Fatigue Syndrome/Myalgic Encephalomyelitis in Australian Patients. Clin. Epidemiol..

[57] Wallis A., Ball M., McKechnie S., Butt H., Lewis D.P., Bruck D. (2017). Examining Clinical Similarities between Myalgic Encephalomyelitis/Chronic Fatigue Syndrome and d-Lactic Acidosis: A Systematic Review. J. Transl. Med..

[58] Corbitt M., Campagnolo N., Staines D., Marshall-Gradisnik S. (2018). A Systematic Review of Probiotic Interventions for Gastrointestinal Symptoms and Irritable Bowel Syndrome in Chronic Fatigue Syndrome/Myalgic Encephalomyelitis (CFS/ME). Probiotics Antimicrob. Proteins.

[59] Riedl A., Schmidtmann M., Stengel A., Goebel M., Wisser A.-S., Klapp B.F., Mönnikes H. (2008). Somatic Comorbidities of Irritable Bowel Syndrome: A Systematic Analysis. J. Psychosom. Res..

[60] Lakhan S.E., Kirchgessner A. (2010). Gut Inflammation in Chronic Fatigue Syndrome. Nutr. Metab..

[61] Mandarano A.H., Giloteaux L., Keller B.A., Levine S.M., Hanson M.R. (2018). Eukaryotes in the Gut Microbiota in Myalgic Encephalomyelitis/Chronic Fatigue Syndrome. PeerJ.

[62] Sheedy J.R., Wettenhall R.E.H., Scanlon D., Gooley P.R., Lewis D.P., McGregor N., Stapleton D.I., Butt H.L., de Meirleir K.L. (2009). Increased D-Lactic Acid Intestinal Bacteria in Patients with Chronic Fatigue Syndrome. In Vivo.

[63] Lupo G.F.D., Rocchetti G., Lucini L., Lorusso L., Manara E., Bertelli M., Puglisi E., Capelli E. (2021). Potential Role of Microbiome in Chronic Fatigue Syndrome/Myalgic Encephalomyelits (CFS/ME). Sci. Rep..

[64] Frémont M., Coomans D., Massart S., de Meirleir K. (2013). High-Throughput 16S RRNA Gene Sequencing Reveals Alterations of Intestinal Microbiota in Myalgic Encephalomyelitis/Chronic Fatigue Syndrome Patients. Anaerobe.

[65] Kitami T., Fukuda S., Kato T., Yamaguti K., Nakatomi Y., Yamano E., Kataoka Y., Mizuno K., Tsuboi Y., Kogo Y. (2020). Deep Phenotyping of Myalgic Encephalomyelitis/Chronic Fatigue Syndrome in Japanese Population. Sci. Rep..

[66] Shukla S.K., Cook D., Meyer J., Vernon S.D., Le T., Clevidence D., Robertson C.E., Schrodi S.J., Yale S., Frank D.N. (2015). Changes in Gut and Plasma Microbiome Following Exercise Challenge in Myalgic Encephalomyelitis/Chronic Fatigue Syndrome (ME/CFS). PLoS ONE.

[67] Manichanh C. (2006). Reduced Diversity of Faecal Microbiota in Crohn’s Disease Revealed by a Metagenomic Approach. Gut.

[68] Hevia A., Milani C., López P., Cuervo A., Arboleya S., Duranti S., Turroni F., González S., Suárez A., Gueimonde M. (2014). Intestinal Dysbiosis Associated with Systemic Lupus Erythematosus. mBio.

[69] Gianchecchi E., Fierabracci A. (2019). Recent Advances on Microbiota Involvement in the Pathogenesis of Autoimmunity. Int. J. Mol. Sci..

[70] Larsen N., Vogensen F.K., van den Berg F.W.J., Nielsen D.S., Andreasen A.S., Pedersen B.K., Al-Soud W.A., Sørensen S.J., Hansen L.H., Jakobsen M. (2010). Gut Microbiota in Human Adults with Type 2 Diabetes Differs from Non-Diabetic Adults. PLoS ONE.

[71] Giloteaux L., Hanson M.R., Keller B.A. (2016). A Pair of Identical Twins Discordant for Myalgic Encephalomyelitis/Chronic Fatigue Syndrome Differ in Physiological Parameters and Gut Microbiome Composition. Am. J. Med. Case Rep..

[72] Holmes G.P. (1988). Chronic Fatigue Syndrome: A Working Case Definition. Ann. Intern. Med..

[73] Balows A., Hausler W., Herrmann K., Isenberg H., Shadomy H. (2007). Manual of Clinical Microbiology.

[74] Maes M., Mihaylova I., Leunis J.C. (2007). Increased Serum IgA and IgM against LPS of Enterobacteria in Chronic Fatigue Syndrome (CFS): Indication for the Involvement of Gram-Negative Enterobacteria in the Etiology of CFS and for the Presence of an Increased Gut-Intestinal Permeability. J. Affect. Disord..

[75] Morris G., Maes M. (2014). Oxidative and Nitrosative Stress and Immune-Inflammatory Pathways in Patients with Myalgic Encephalomyelitis (ME)/Chronic Fatigue Syndrome (CFS). Curr. Neuropharmacol..

[76] Maes M., Twisk F.N.M., Kubera M., Ringel K., Leunis J.C., Geffard M. (2012). Increased IgA Responses to the LPS of Commensal Bacteria Is Associated with Inflammation and Activation of Cell-Mediated Immunity in Chronic Fatigue Syndrome. J. Affect. Disord..

[77] Maes M., Leunis J.-C. (2008). Normalization of Leaky Gut in Chronic Fatigue Syndrome (CFS) Is Accompanied by a Clinical Improvement: Effects of Age, Duration of Illness and the Translocation of LPS from Gram-Negative Bacteria. Neuro Endocrinol. Lett..

[78] Malla M.A., Dubey A., Kumar A., Yadav S., Hashem A., Abd_Allah E.F. (2019). Exploring the Human Microbiome: The Potential Future Role of Next-Generation Sequencing in Disease Diagnosis and Treatment. Front. Immunol..

[79] Lemke D., Klement R.J., Schweiger F., Schweiger B., Spitz J. (2021). Vitamin D Resistance as a Possible Cause of Autoimmune Diseases: A Hypothesis Confirmed by a Therapeutic High-Dose Vitamin D Protocol. Front. Immunol..

[80] Komaroff M.A.L., Buchwald M.D.S. (1998). CHRONIC FATIGUE SYNDROME: An Update. Annu. Rev. Med..

[81] Du Preez S., Corbitt M., Cabanas H., Eaton N., Staines D., Marshall-Gradisnik S. (2018). A Systematic Review of Enteric Dysbiosis in Chronic Fatigue Syndrome/Myalgic Encephalomyelitis. Syst. Rev..

[82] Groschwitz K.R., Hogan S.P. (2009). Intestinal Barrier Function: Molecular Regulation and Disease Pathogenesis. J. Allergy Clin. Immunol..

[83] Alhasson F., Das S., Seth R., Dattaroy D., Chandrashekaran V., Ryan C.N., Chan L.S., Testerman T., Burch J., Hofseth L.J. (2017). Altered Gut Microbiome in a Mouse Model of Gulf War Illness Causes Neuroinflammation and Intestinal Injury via Leaky Gut and TLR4 Activation. PLoS ONE.

[84] Mohammad S., Thiemermann C. (2021). Role of Metabolic Endotoxemia in Systemic Inflammation and Potential Interventions. Front. Immunol..

[85] Lucas K., Maes M. (2013). Role of the Toll like Receptor (TLR) Radical Cycle in Chronic Inflammation: Possible Treatments Targeting the TLR4 Pathway. Mol. Neurobiol..

[86] Munford R. (2016). Endotoxemia-Menace, Marker, or Mistake?. J. Leukoc. Biol..

[87] Maes M., Kubera M., Leunis J.C., Berk M. (2012). Increased IgA and IgM Responses against Gut Commensals in Chronic Depression: Further Evidence for Increased Bacterial Translocation or Leaky Gut. J. Affect. Disord..

[88] Maes M., Mihaylova I., Kubera M., Leunis J. (2008). An IgM-Mediated Immune Response Directed against Nitro-Bovine Serum Albumin (Nitro-BSA) in Chronic Fatigue Syndrome (CFS) and Major Depression: Evidence That Nitrosative Stress Is Another Factor Underpinning the Comorbidity between Major Depression and CFS. Neuro Endocrinol. Lett..

[89] Sartori A.C., Vance D.E., Slater L.Z., Crowe M. (2012). The Impact of Inflammation on Cognitive Function in Older Adults: Implications for Healthcare Practice and Research. J. Neurosci. Nurs..

[90] Gorelick P.B. (2010). Role of Inflammation in Cognitive Impairment: Results of Observational Epidemiological Studies and Clinical Trials. Ann. N.Y. Acad. Sci..

[91] Slyepchenko A., Maes M., Jacka F.N., Köhler C.A., Barichello T., McIntyre R.S., Berk M., Grande I., Foster J.A., Vieta E. (2016). Gut Microbiota, Bacterial Translocation, and Interactions with Diet: Pathophysiological Links between Major Depressive Disorder and Non-Communicable Medical Comorbidities. Psychother. Psychosom..

[92] Morris G., Maes M., Berk M., Puri B.K. (2019). Myalgic Encephalomyelitis or Chronic Fatigue Syndrome: How Could the Illness Develop?. Metab. Brain Dis..

[93] Sotzny F., Blanco J., Capelli E., Castro-Marrero J., Steiner S., Murovska M., Scheibenbogen C. (2018). Myalgic Encephalomyelitis/Chronic Fatigue Syndrome—Evidence for an Autoimmune Disease. Autoimmun. Rev..

[94] Morris G., Berk M., Carvalho A., Caso J., Sanz Y., Maes M. (2016). The Role of Microbiota and Intestinal Permeability in the Pathophysiology of Autoimmune and Neuroimmune Processes with an Emphasis on Inflammatory Bowel Disease Type 1 Diabetes and Chronic Fatigue Syndrome. Curr. Pharm. Des..

[95] Morris G., Berk M., Galecki P., Maes M. (2014). The Emerging Role of Autoimmunity in Myalgic Encephalomyelitis/Chronic Fatigue Syndrome (ME/Cfs). Mol. Neurobiol..

[96] Schieber M., Chandel N.S. (2014). ROS Function in Redox Signaling and Oxidative Stress. Curr. Biol..

[97] Sido B., Hack V., Hochlehnert A., Lipps H., Herfarth C., Dröge W. (1998). Impairment of Intestinal Glutathione Synthesis in Patients with Inflammatory Bowel Disease. Gut.

[98] Morris G., Puri B.K., Walker A.J., Maes M., Carvalho A.F., Walder K., Mazza C., Berk M. (2019). Myalgic Encephalomyelitis/Chronic Fatigue Syndrome: From Pathophysiological Insights to Novel Therapeutic Opportunities. Pharmacol. Res..

[99] Fukuda S., Nojima J., Motoki Y., Yamaguti K., Nakatomi Y., Okawa N., Fujiwara K., Watanabe Y., Kuratsune H. (2016). A Potential Biomarker for Fatigue: Oxidative Stress and Anti-Oxidative Activity. Biol. Psychol..

[100] Maes M., Mihaylova I., Kubera M., Uytterhoeven M., Vrydags N., Bosmans E. (2009). Increased 8-Hydroxy-Deoxyguanosine, a Marker of Oxidative Damage to DNA, in Major Depression and Myalgic Encephalomyelitis/Chronic Fatigue Syndrome. Neuro Endocrinol. Lett..

[101] Monro J.A., Puri B.K. (2018). A Molecular Neurobiological Approach to Understanding the Aetiology of Chronic Fatigue Syndrome (Myalgic Encephalomyelitis or Systemic Exertion Intolerance Disease) with Treatment Implications. Mol. Neurobiol..

[102] Ivashkin V., Poluektov Y., Kogan E., Shifrin O., Sheptulin A., Kovaleva A., Kurbatova A., Krasnov G., Poluektova E. (2021). Disruption of the Pro-Inflammatory, Anti-Inflammatory Cytokines and Tight Junction Proteins Expression, Associated with Changes of the Composition of the Gut Microbiota in Patients with Irritable Bowel Syndrome. PLoS ONE.

[103] Raijmakers R.P.H., Roerink M.E., Jansen A.F.M., Keijmel S.P., Gacesa R., Li Y., Joosten L.A.B., van der Meer J.W.M., Netea M.G., Bleeker-Rovers C.P. (2020). Multi-Omics Examination of Q Fever Fatigue Syndrome Identifies Similarities with Chronic Fatigue Syndrome. J. Transl. Med..

[104] Maes M. (2015). A New Case Definition of Neuro-Inflammatory and Oxidative Fatigue (NIOF), a Neuroprogressive Disorder, Formerly Known as Chronic Fatigue Syndrome or Myalgic Encephalomyelitis: Results of Multivariate Pattern Recognition Methods and External Validation by Neuro-Immune Biomarkers. Neuro Endocrinol. Lett..

[105] Borton M.A., Sabag-Daigle A., Wu J., Solden L.M., O’Banion B.S., Daly R.A., Wolfe R.A., Gonzalez J.F., Wysocki V.H., Ahmer B.M.M. (2017). Chemical and Pathogen-Induced Inflammation Disrupt the Murine Intestinal Microbiome. Microbiome.

[106] Lee J.S., Kim H.G., Lee D.S., Son C.G. (2018). Oxidative Stress Is a Convincing Contributor to Idiopathic Chronic Fatigue. Sci. Rep..

[107] Maes M., Twisk F. (2009). Why Myalgic Encephalomyelitis/Chronic Fatigue Syndrome (ME/CFS) May Kill You: Disorders in the Inflammatory and Oxidative and Nitrosative Stress (IO&NS) Pathways May Explain Cardiovascular Disorders in ME/CFS. Neuro Endocrinol. Lett..

[108] Logan A.C., Rao A.V., Irani D. (2003). Chronic Fatigue Syndrome: Lactic Acid Bacteria May Be of Therapeutic Value. Med. Hypotheses.

[109] Sullivan Å., Nord C.E., Evengård B. (2009). Effect of Supplement with Lactic-Acid Producing Bacteria on Fatigue and Physical Activity in Patients with Chronic Fatigue Syndrome. Nutr. J..

[110] Campagnolo N., Johnston S., Collatz A., Staines D., Marshall-Gradisnik S. (2017). Dietary and Nutrition Interventions for the Therapeutic Treatment of Chronic Fatigue Syndrome/Myalgic Encephalomyelitis: A Systematic Review. J. Hum. Nutr. Diet..

[111] Staudacher H.M., Lomer M.C.E., Farquharson F.M., Louis P., Fava F., Franciosi E., Scholz M., Tuohy K.M., Lindsay J.O., Irving P.M. (2017). A Diet Low in FODMAPs Reduces Symptoms in Patients With Irritable Bowel Syndrome and A Probiotic Restores Bifidobacterium Species: A Randomized Controlled Trial. Gastroenterology.

[112] Hod K., Sperber A.D., Ron Y., Boaz M., Dickman R., Berliner S., Halpern Z., Maharshak N., Dekel R. (2017). A Double-Blind, Placebo-Controlled Study to Assess the Effect of a Probiotic Mixture on Symptoms and Inflammatory Markers in Women with Diarrhea-Predominant IBS. Neurogastroenterol. Motil..

[113] Ishaque S.M., Khosruzzaman S.M., Ahmed D.S., Sah M.P. (2018). A Randomized Placebo-Controlled Clinical Trial of a Multi-Strain Probiotic Formulation (Bio-Kult^®^) in the Management of Diarrhea-Predominant Irritable Bowel Syndrome. BMC Gastroenterol..

[114] Francavilla R., Piccolo M., Francavilla A., Polimeno L., Semeraro F., Cristofori F., Castellaneta S., Barone M., Indrio F., Gobbetti M. (2019). Clinical and Microbiological Effect of a Multispecies Probiotic Supplementation in Celiac Patients with Persistent IBS-Type Symptoms: A Randomized, Double-Blind, Placebo-Controlled, Multicenter Trial. J. Clin. Gastroenterol..

[115] Leventogiannis K., Gkolfakis P., Spithakis G., Tsatali A., Pistiki A., Sioulas A., Giamarellos-Bourboulis E.J., Triantafyllou K. (2019). Effect of a Preparation of Four Probiotics on Symptoms of Patients with Irritable Bowel Syndrome: Association with Intestinal Bacterial Overgrowth. Probiotics Antimicrob. Proteins.

[116] Oh J.H., Jang Y.S., Kang D., Chang D.K., Min Y.W. (2019). Efficacy and Safety of New Lactobacilli Probiotics for Unconstipated Irritable Bowel Syndrome: A Randomized, Double-Blind, Placebo-Controlled Trial. Nutrients.

[117] Lewis E.D., Antony J.M., Crowley D.C., Piano A., Bhardwaj R., Tompkins T.A., Evans M. (2020). Efficacy of Lactobacillus Paracasei Ha-196 and Bifidobacterium Longum R0175 in Alleviating Symptoms of Irritable Bowel Syndrome (IBS): A Randomized, Placebo-Controlled Study. Nutrients.

[118] Lorenzo-Zúñiga V., Llop E., Suárez C., Álvarez B., Abreu L., Espadaler J., Serra J. (2014). I. 31, a New Combination of Probiotics, Improves Irritable Bowel Syndrome-Related Quality of Life. World J. Gastroenterol..

[119] Skrzydło-Radomańska B., Prozorow-Król B., Cichoż-Lach H., Majsiak E., Bierła J.B., Kosikowski W., Szczerbiński M., Gantzel J., Cukrowska B. (2020). The Effectiveness of Synbiotic Preparation Containing Lactobacillus and Bifidobacterium Probiotic Strains and Short Chain Fructooligosaccharides in Patients with Diarrhea Predominant Irritable Bowel Syndrome—a Randomized Double-Blind, Placebo-Controlled Study. Nutrients.

[120] Pinto-Sanchez M., Hall G., Ghajar K., Nardelli A., Bolino C., Lau J., Martin F., Cominetti O., Welsh C., Rieder A. (2017). Probiotic Bifidobacterium Longum NCC3001 Reduces Depression Scores and Alters Brain Activity: A Pilot Study in Patients With Irritable Bowel Syndrome. Gastroenterology.

[121] Yuan F., Ni H., Asche C., Kim M., Walayat S., Ren J. (2017). Efficacy of Bifidobacterium Infantis 35624 in Patients with Irritable Bowel Syndrome: A Meta-Analysis. Curr. Med. Res. Opin..

[122] Andresen V., Gschossmann J., Layer P. (2020). Heat-Inactivated Bifidobacterium Bifidum MIMBb75 (SYN-HI-001) in the Treatment of Irritable Bowel Syndrome: A Multicentre, Randomised, Double-Blind, Placebo-Controlled Clinical Trial. Lancet Gastroenterol. Hepatol..

[123] Zhao Q., Yang W.R., Wang X.H., Li G.Q., Xu L.Q., Cui X., Liu Y., Zuo X.L. (2019). Clostridium Butyricum Alleviates Intestinal Low-Grade Inflamm TNBS-Induced Irritable Bowel Syndrome in Mice by Regulating Functional Status of Lamina Propria Dendritic Cells. World J. Gastroenterol..

[124] Basturk A., Artan R., Yilmaz A. (2020). Efficacy of Synbiotic, Probiotic, and Prebiotic Treatments for Irritable Bowel Syndrome in Children: A Randomized Controlled Trial. Turk. J. Gastroenterol..

[125] Chelakkot C., Choi Y., Kim D.K., Park H.T., Ghim J., Kwon Y., Jeon J., Kim M.S., Jee Y.K., Gho Y.S. (2018). Akkermansia Muciniphila-Derived Extracellular Vesicles Influence Gut Permeability through the Regulation of Tight Junctions. Exp. Mol. Med..

[126] Lim S.M., Jeong J.J., Woo K.H., Han M.J., Kim D.H. (2016). Lactobacillus Sakei OK67 Ameliorates High-Fat Diet-Induced Blood Glucose Intolerance and Obesity in Mice by Inhibiting Gut Microbiota Lipopolysaccharide Production and Inducing Colon Tight Junction Protein Expression. Nutr. Res..

[127] Venturini L., Bacchi S., Capelli E., Lorusso L., Ricevuti G., Cusa C. (2019). Modification of Immunological Parameters, Oxidative Stress Markers, Mood Symptoms, and Well-Being Status in CFS Patients after Probiotic Intake: Observations from a Pilot Study. Oxid. Med. Cell. Longev..

[128] Groeger D., O’Mahony L., Murphy E.F., Bourke J.F., Dinan T.G., Kiely B., Shanahan F., Quigley E.M.M. (2013). Bifidobacterium Infantis 35624 Modulates Host Inflammatory Processes beyond the Gut. Gut Microbes.

[129] Caswell A., Daniels J. (2018). Anxiety and Depression in Chronic Fatigue Syndrome: Prevalence and Effect on Treatment. A Systematic Review, Meta-Analysis and Meta-Regression.

[130] Karakula-Juchnowicz H., Rog J., Juchnowicz D., Łoniewski I., Skonieczna-Ydecka K., Krukow P., Futyma-Jedrzejewska M., Kaczmarczyk M. (2019). The Study Evaluating the Effect of Probiotic Supplementation on the Mental Status, Inflammation, and Intestinal Barrier in Major Depressive Disorder Patients Using Gluten-Free or Gluten-Containing Diet (SANGUT Study): A 12-Week, Randomized, Double-Blind, and Placebo-Controlled Clinical Study Protocol. Nutr. J..

[131] Griffith J., Zarrouf F. (2008). A Systematic Review of Chronic Fatigue Syndrome: Don’t Assume It’s Depression. Prim. Care Companion J. Clin. Psychiatry.

[132] Rao A.V., Bested A.C., Beaulne T.M., Katzman M.A., Iorio C., Berardi J.M., Logan A.C. (2009). A Randomized, Double-Blind, Placebo-Controlled Pilot Study of a Probiotic in Emotional Symptoms of Chronic Fatigue Syndrome. Gut Pathog..

[133] Davani-Davari D., Negahdaripour M., Karimzadeh I., Seifan M., Mohkam M., Masoumi S.J., Berenjian A., Ghasemi Y. (2019). Prebiotics: Definition, Types, Sources, Mechanisms, and Clinical Applications. Foods.

[134] Den Besten G., van Eunen K., Groen A.K., Venema K., Reijngoud D.J., Bakker B.M. (2013). The Role of Short-Chain Fatty Acids in the Interplay between Diet, Gut Microbiota, and Host Energy Metabolism. J. Lipid Res..

[135] Boudry G., Hamilton M.K., Chichlowski M., Wickramasinghe S., Barile D., Kalanetra K.M., Mills D.A., Raybould H.E. (2017). Bovine Milk Oligosaccharides Decrease Gut Permeability and Improve Inflammation and Microbial Dysbiosis in Diet-Induced Obese Mice. J. Dairy Sci..

[136] Yu T., Wang Y., Chen X., Xiong W., Tang Y., Lin L. (2020). Spirulina Platensis Alleviates Chronic Inflammation with Modulation of Gut Microbiota and Intestinal Permeability in Rats Fed a High-Fat Diet. J. Cell. Mol. Med..

[137] Zhang Z., Lin T., Meng Y., Hu M., Shu L., Jiang H., Gao R., Ma J., Wang C., Zhou X. (2021). FOS/GOS Attenuates High-Fat Diet Induced Bone Loss via Reversing Microbiota Dysbiosis, High Intestinal Permeability and Systemic Inflammation in Mice. Metab. Clin. Exp..

[138] Cani P., Possemiers S., van de Wiele T., Guiot Y., Everard A., Rottier O., Geurts L., Naslain D., Neyrinck A., Lambert L. (2009). Changes in Gut Microbiota Control Inflammation in Obese Mice through a Mechanism Involving GLP-2-Driven Improvement of Gut Permeability. Gut.

[139] Nettleton J.E., Klancic T., Schick A., Choo A.C., Shearer J., Borgland S.L., Chleilat F., Mayengbam S., Reimer R.A. (2019). Low-Dose Stevia (Rebaudioside A) Consumption Perturbs Gut Microbiota and the Mesolimbic Dopamine Reward System. Nutrients.

[140] David L.A., Maurice C.F., Carmody R.N., Gootenberg D.B., Button J.E., Wolfe B.E., Ling A.V., Devlin A.S., Varma Y., Fischbach M.A. (2014). Diet Rapidly and Reproducibly Alters the Human Gut Microbiome. Nature.

[141] Klingbeil E., De C.B., Serre L. (2018). Microbiota Modulation by Eating Patterns and Diet Composition: Impact on Food Intake. Am. J. Physiol. Regul. Integr. Comp. Physiol..

[142] Merra G., Noce A., Marrone G., Cintoni M., Tarsitano M.G., Capacci A., de Lorenzo A. (2021). Influence of Mediterranean Diet on Human Gut Microbiota. Nutrients.

[143] El-Salhy M., Hatlebakk J.G., Hausken T. (2019). Diet in Irritable Bowel Syndrome (IBS): Interaction with Gut Microbiota and Gut Hormones. Nutrients.

[144] Varjú P., Farkas N., Hegyi P., Garami A., Szabó I., Illés A., Solymár M., Vincze Á., Balaskó M., Pár G. (2017). Low Fermentable Oligosaccharides, Disaccharides, Monosaccharides and Polyols (FODMAP) Diet Improves Symptoms in Adults Suffering from Irritable Bowel Syndrome (IBS) Compared to Standard IBS Diet: A Meta-Analysis of Clinical Studies. PLoS ONE.

[145] Cuomo R., Andreozzi P., Zito F.P., Passananti V., de Carlo G., Sarnelli G. (2014). Irritable Bowel Syndrome and Food Interaction. World J. Gastroenterol..

[146] Singh R.K., Chang H.W., Yan D., Lee K.M., Ucmak D., Wong K., Abrouk M., Farahnik B., Nakamura M., Zhu T.H. (2017). Influence of Diet on the Gut Microbiome and Implications for Human Health. J. Transl. Med..

[147] Liu R., Hong J., Xu X., Feng Q., Zhang D., Gu Y., Shi J., Zhao S., Liu W., Wang X. (2017). Gut Microbiome and Serum Metabolome Alterations in Obesity and after Weight-Loss Intervention. Nat. Med..

[148] Suskind D.L., Lee D., Kim Y.M., Wahbeh G., Singh N., Braly K., Nuding M., Nicora C.D., Purvine S.O., Lipton M.S. (2020). The Specific Carbohydrate Diet and Diet Modification as Induction Therapy for Pediatric Crohn’s Disease: A Randomized Diet Controlled Trial. Nutrients.

[149] Marx W., Moseley G., Berk M., Jacka F. (2017). Nutritional Psychiatry: The Present State of the Evidence. Proc. Nutr. Soc..

[150] Snelson M., Clarke R.E., Nguyen T., Penfold S.A., Forbes J.M., Tan S.M., Coughlan M.T. (2021). Long Term High Protein Diet Feeding Alters the Microbiome and Increases Intestinal Permeability, Systemic Inflammation and Kidney Injury in Mice. Mol. Nutr. Food Res..

[151] Do M., Lee E., Oh M.-J., Kim Y., Park H.-Y. (2018). High-Glucose or -Fructose Diet Cause Changes of the Gut Microbiota and Metabolic Disorders in Mice without Body Weight Change. Nutrients.

[152] Nilholm C., Roth B., Ohlsson B. (2019). A Dietary Intervention with Reduction of Starch and Sucrose Leads to Reduced Gastrointestinal and Extra-Intestinal Symptoms in IBS Patients. Nutrients.

[153] Ott B., Skurk T., Hastreiter L., Lagkouvardos I., Fischer S., Büttner J., Kellerer T., Clavel T., Rychlik M., Haller D. (2017). Effect of Caloric Restriction on Gut Permeability, Inflammation Markers, and Fecal Microbiota in Obese Women. Sci. Rep..

[154] Kaliannan K., Wang B., Li X.Y., Kim K.J., Kang J.X. (2015). A Host-Microbiome Interaction Mediates the Opposing Effects of Omega-6 and Omega-3 Fatty Acids on Metabolic Endotoxemia. Sci. Rep..

[155] Puri B.K. (2004). The Use of Eicosapentaenoic Acid in the Treatment of Chronic Fatigue Syndrome. Prostaglandins Leukot. Essent. Fat. Acids.

[156] Puri B.K. (2007). Long-Chain Polyunsaturated Fatty Acids and the Pathophysiology of Myalgic Encephalomyelitis (Chronic Fatigue Syndrome). J. Clin. Pathol..

[157] Wang J.H., Bose S., Kim G.C., Hong S.U., Kim J.H., Kim J.E., Kim H. (2014). Flos Lonicera Ameliorates Obesity and Associated Endotoxemia in Rats through Modulation of Gut Permeability and Intestinal Microbiota. PLoS ONE.

[158] Wang S., Li Q., Zang Y., Zhao Y., Liu N., Wang Y., Xu X., Liu L., Mei Q. (2017). Apple Polysaccharide Inhibits Microbial Dysbiosis and Chronic Inflammation and Modulates Gut Permeability in HFD-Fed Rats. Int. J. Biol. Macromol..

[159] Fujisaka S., Usui I., Nawaz A., Igarashi Y., Okabe K., Furusawa Y., Watanabe S., Yamamoto S., Sasahara M., Watanabe Y. (2020). Bofutsushosan Improves Gut Barrier Function with a Bloom of Akkermansia Muciniphila and Improves Glucose Metabolism in Mice with Diet-Induced Obesity. Sci. Rep..

[160] Wang X., Qu Y., Zhang Y., Li S., Sun Y., Chen Z., Teng L., Wang D. (2018). Antifatigue Potential Activity of Sarcodon Imbricatus in Acute Excise-Treated and Chronic Fatigue Syndrome in Mice via Regulation of Nrf2-Mediated Oxidative Stress. Oxidative Med. Cell. Longev..

[161] Kwon D.A., Kim Y.S., Kim S.K., Baek S.H., Kim H.K., Lee H.S. (2021). Antioxidant and Antifatigue Effect of a Standardized Fraction (HemoHIM) from Angelica Gigas, Cnidium Officinale, and Paeonia Lactiflora. Pharm. Biol..

[162] Tan P., Li X., Shen J., Feng Q. (2020). Fecal Microbiota Transplantation for the Treatment of Inflammatory Bowel Disease: An Update. Front. Pharmacol..

[163] Gupta A., Khanna S. (2017). Fecal Microbiota Transplantation. JAMA.

[164] Rodiño-Janeiro B.K., Vicario M., Alonso-Cotoner C., Pascua-García R., Santos J. (2018). A Review of Microbiota and Irritable Bowel Syndrome: Future in Therapies. Adv. Ther..

[165] Surawicz C.M., Brandt L.J., Binion D.G., Ananthakrishnan A.N., Curry S.R., Gilligan P.H., McFarland L.V., Mellow M., Zuckerbraun B.S. (2013). Guidelines for Diagnosis, Treatment, and Prevention of Clostridium Difficile Infections. Am. J. Gastroenterol..

[166] Malnick S.D.H., Fisher D., Somin M., Neuman M.G. (2021). Treating the Metabolic Syndrome by Fecal Transplantation—Current Status. Biology.

[167] Choi H.H., Cho Y.S. (2016). Fecal Microbiota Transplantation: Current Applications, Effectiveness, and Future Perspectives. Clin. Endosc..

[168] Juul F.E., Garborg K., Bretthauer M., Skudal H., Øines M.N., Wiig H., Rose Ø., Seip B., Lamont J.T., Midtvedt T. (2018). Fecal Microbiota Transplantation for Primary Clostridium Difficile Infection. N. Engl. J. Med..

[169] Chen D., Wu J., Jin D., Wang B., Cao H. (2019). Fecal Microbiota Transplantation in Cancer Management: Current Status and Perspectives. Int. J. Cancer.

[170] Evrensel A., Ceylan M.E. (2016). Fecal Microbiota Transplantation and Its Usage in Neuropsychiatric Disorders. Clin. Psychopharmacol. Neurosci..

[171] Xu M.Q., Cao H.L., Wang W.Q., Wang S., Cao X.C., Yan F., Wang B.M. (2015). Fecal Microbiota Transplantation Broadening Its Application beyond Intestinal Disorders. World J. Gastroenterol..

[172] Kim M., Kim Y., Choi H., Kim W., Park S., Lee D., Kim D., Kim H., Choi H., Hyun D. (2020). Transfer of a Healthy Microbiota Reduces Amyloid and Tau Pathology in an Alzheimer’s Disease Animal Model. Gut.

[173] Wang Y., Wiesnoski D.H., Helmink B.A., Gopalakrishnan V., Choi K., DuPont H.L., Jiang Z.D., Abu-Sbeih H., Sanchez C.A., Chang C.C. (2018). Fecal Microbiota Transplantation for Refractory Immune Checkpoint Inhibitor-Associated Colitis. Nat. Med..

[174] Shen Z.-H., Zhu C.-X., Quan Y.-S., Yang Z.-Y., Wu S., Luo W.-W., Tan B., Wang X.-Y. (2018). Relationship between Intestinal Microbiota and Ulcerative Colitis: Mechanisms and Clinical Application of Probiotics and Fecal Microbiota Transplantation. World J. Gastroenterol..

[175] Borody T. (1995). Bacteriotherapy for Chronic Fatigue Syndrome: A Long-Term Follow up Study. Proceedings of the 1995 CFS National Consensus Conference.

[176] Borody T.J., Nowak A., Finlayson S. (2012). The GI Microbiome and Its Role in Chronic Fatigue Syndrome: A Summary of BacteriotherapyThe GI Microbiome and Its Role in Chronic Fatigue Syndrome: A Summary of Bacteriotherapy. ACNEM J..

[177] Schmulson M., Bashashati M. (2018). Fecal Microbiota Transfer for Bowel Disorders: Efficacy or Hype?. Curr. Opin. Pharmacol..

[178] Lopetuso L., Ianiro G., Allegretti J., Bibbò S., Gasbarrini A., Scaldaferri F., Cammarota G. (2020). Fecal Transplantation for Ulcerative Colitis: Current Evidence and Future Applications. Expert Opin. Biol. Ther..

[179] Shanahan F., Quigley E. (2014). Manipulation of the Microbiota for Treatment of IBS and IBD-Challenges and Controversies. Gastroenterology.

[180] Imdad A., Nicholson M., Tanner-Smith E., Zackular Z., Gomez-Duarte O., Beaulieu D., Acra S. (2018). Fecal Transplantation for Treatment of Inflammatory Bowel Disease. Cochrane Database Syst. Rev..

[181] Aroniadis O.C., Brandt L.J. (2013). Fecal Microbiota Transplantation: Past, Present and Future. Curr. Opin. Gastroenterol..

[182] Levy A.N., Allegretti J.R. (2019). Insights into the Role of Fecal Microbiota Transplantation for the Treatment of Inflammatory Bowel Disease. Ther. Adv. Gastroenterol..

